# *SLC25A13* Gene Analysis in Citrin Deficiency: Sixteen Novel Mutations in East Asian Patients, and the Mutation Distribution in a Large Pediatric Cohort in China

**DOI:** 10.1371/journal.pone.0074544

**Published:** 2013-09-19

**Authors:** Yuan-Zong Song, Zhan-Hui Zhang, Wei-Xia Lin, Xin-Jing Zhao, Mei Deng, Yan-Li Ma, Li Guo, Feng-Ping Chen, Xiao-Ling Long, Xiang-Ling He, Yoshihide Sunada, Shun Soneda, Akiko Nakatomi, Sumito Dateki, Lock-Hock Ngu, Keiko Kobayashi, Takeyori Saheki

**Affiliations:** 1 Department of Pediatrics, The First Affiliated Hospital, Jinan University, Guangzhou, China; 2 Department of Laboratory Science, The First Affiliated Hospital, Jinan University, Guangzhou, China; 3 Department of Pediatrics, Zhongshan Boai Hospital, Zhongshan, China; 4 Children Medical Center, Hunan Provincial People’s Hospital, Changsha, China; 5 Department of Neurology, Kawasaki Medical School, Kawasaki, Japan; 6 Department of Pediatrics, St. Marianna University School of Medicine, Kanagawa, Japan; 7 Department of Pediatrics, Nagasaki University Hospital, Nagasaki, Japan; 8 Clinical Genetic Department, Hospital Kuala Lumpur, Jalan Pahang, Kuala Lumpur, Malaysia; 9 Department of Molecular Metabolism and Biochemical Genetics, Kagoshima University Graduate School of Medical and Dental Sciences, Kagoshima, Japan; 10 Institute of Resource Development and Analysis, Kumamoto University, Kumamoto, Japan; Vanderbilt University Medical Center, United States of America

## Abstract

**Background:**

The human *SLC25A13* gene encodes citrin, the liver-type mitochondrial aspartate/glutamate carrier isoform 2 (AGC2), and *SLC25A13* mutations cause citrin deficiency (CD), a disease entity that encompasses different age-dependant clinical phenotypes such as Adult-onset Citrullinemia Type II (CTLN2) and Neonatal Intrahepatic Cholestasis caused by Citrin Deficiency (NICCD). The analyses of *SLC25A13* gene and its protein/mRNA products remain reliable tools for the definitive diagnoses of CD patients, and so far, the *SLC25A13* mutation spectrum in Chinese CD patients has not been well-characterized yet.

**Methods and Results:**

By means of direct DNA sequencing, cDNA cloning and SNP analyses, 16 novel pathogenic mutations, including 9 missense, 4 nonsense, 1 splice-site, 1 deletion and 1 large transposal insertion IVS4ins6kb (GenBank accession number KF425758), were identified in CTLN2 or NICCD patients from China, Japan and Malaysia, respectively, making the *SLC25A13* variations worldwide reach the total number of 81. A large NICCD cohort of 116 Chinese cases was also established, and the 4 high-frequency mutations contributed a much larger proportion of the mutated alleles in the patients from south China than in those from the north (χ^2^ = 14.93, *P*<0.01), with the latitude of 30°N as the geographic dividing line in mainland China.

**Conclusions:**

This paper further enriched the *SLC25A13* variation spectrum worldwide, and formed a substantial contribution to the in-depth understanding of the genotypic feature of Chinese CD patients.

## Introduction

The term CITRIN was designated in 1999 to stand for the protein product encoded by *SLC25A13* gene, which was localized to chromosome 7q21.3 and cloned as the causative gene for Adult-onset Citrullinemia Type 2 (CTLN2, OMIM #603471) [1]. *SLC25A13* mutations result in citrin deficiency (CD), and CTLN2 was the firstly-described CD phenotype, which occurs in adolescents or adults and the prognosis is usually not benign [2]. Subsequently, CD was found to be associated with intrahepatic cholestasis in neonates or infants [3]–[5], and these findings led to the introduction of a new nomenclature of Neonatal Intrahepatic Cholestasis caused by Citrin Deficiency (NICCD, OMIM #605814) [6]–[8]. NICCD usually demonstrates satisfactory clinical outcomes, with the clinical and laboratory presentations resolving in the first year of life. It has been traditionally-assumed for years that there is a “silent” or “apparently-healthy” stage after the NICCD period but before CTLN2 onset. However, this concept has been challenged by accumulating clinical, laboratory and even behavioral evidences [9]–[11], and actually, an additional CD phenotype, Failure to Thrive and Dyslipidemia caused by Citrin Deficiency (FTTDCD), has been put forward recently [2], [12]–[14].

The phenotypic features of CD patients are really complicated indeed, involving numerous clinical, biochemical, imaging, hepatohistological and metabolomic alterations [13], [15]–[18]. However, none of these changes are pathognomonic, and *SLC25A13* genetic analysis has been recognized as a reliable method for the definitive diagnosis of CD. In the case of unknown mutation after the conventional genetic analyses such as PCR-RFLP and direct DNA sequencing, detection of the protein and mRNA product of *SLC25A13* gene could be considered as the alternative diagnostic tools. However, cultured fibroblasts or biopsied/autopsied liver specimens are usually needed in such analyses [1], [19]–[23], and obtaining these invasive samples may not always be feasible. To address this issue, citrin protein analysis using peripheral blood lymphocytes (PBLs) had been tried [24]. More recently, it was proved that *SLC25A13* cDNA cloning analysis using human PBLs could be taken as a less invasive and more feasible tool to identify aberrant *SLC25A13* transcripts for the molecular diagnosis of CD [14]. However, more experience should be accumulated on the application of this new approach in identifying unknown *SLC25A13* mutations.

Owing to the establishment of the molecular diagnostic tools, increasing number of CD patients were diagnosed in Asia [25]–[34], North America [20], [21], [35], and Europe [36]–[38]. Nevertheless, up to now, the majority of the reported CD patients were from East Asian countries, especially Japan [1], [3]–[5], [8], [9], [11], [15], [16], [22], [24], [39]–[43]. China is a vast country with a huge population. According to the latest official data (http://www.chinapop.gov.cn/xwzx/rkxw/), the resident population in China is more than 1.3 billion, with 472 millions of them in the south area of Yangtze River. The carrier rates of *SLC25A13* mutations had been documented to be 1/63 in China, and particularly 1/48 in its south area [44], [45]. Therefore, if calculated according to Hardy-Weinberg principle, the number of CD patients in China would be approximately 85 700, over 51 300 of whom are living in the South China. However, the officially-reported CD patients from China, including those from the south, were rather limited in number [13], [14], [17], [23], [30], [32], [33], and the distribution of *SLC25A13* mutations in Chinese patients remains far from being completely elucidated.

In this paper, novel *SLC25A13* mutations in 16 Asian CD patients were identified, including a large transposal insertion that gave rise to exon 5 skipping in *SLC25A13* transcripts. We established a large pediatric cohort of Chinese CD patients, and then explored the mutation spectrum and compared the distribution in different geographical areas. We herein reported the findings.

## Subjects and Methods

### Subjects

The research subjects in this paper encompassed 68 new CD patients comprising 60 Chinese, 7 Japanese and 1 Malaysian. In order to explore the *SLC25A13* mutation spectrum and the distribution feature in different areas of China, 56 CD patients reported previously by our group were also enrolled in this paper to establish a large pediatric CD cohort.

### Ethics Statement

This study has been approved by the Committee for Ethics of Kagoshima University Faculty of Medicine, Japan, and by the Medical Ethical Committee, the First Affiliated Hospital, Jinan University, China, and adheres to the World Medical Association Declaration of Helsinki (WMADH 2008), which was adopted by the 59th WMA General Assembly, Seoul, in October 2008. *SLC25A13* analyses were conducted with the written informed consents from the patients or their guardians.

### Mutation Screening and Direct DNA Sequencing

All patients were initially screened for the frequently recurring *SLC25A13* mutations. Thirteen *SLC25A13* mutations were included in the screening panel for the 7 Japanese patients in Kagoshima University, Japan, as in reference [22]. And, 4 mutations were screened in the remaining 61 novel subjects in Jinan University, China. In the subjects whose initial screening only revealed one mutated allele, the 18 exons and their flanking sequences in *SLC25A13* gene were analyzed by direct DNA sequencing, using the approach as described in our previous publications [13], [14], [17], [33].

### Pathogenicity Analysis of the Novel Mutations

According to the HUGO mutation database initiative/Human Genome Variation Society (Instruction for Authors [DB/OL]. New Jersey (IL): John Wiley & Sons, Inc. 2008 [2008-03-24]. http://www3.interscience.wiley.com/journal/38515/home/ForAuthors.html), proof of pathogenicity in this study was defined by at least one of the following criteria: (1) a mutation presenting at the frequency of <1% in at least 50 control individuals, (2) a mutation with co-segregation in a family; (3) alteration of an evolutionary conserved amino acid residue, and (4) nonsense and deletion variation in the coding sequence of *SLC25A13* gene. Moreover, the function effect of the novel missense mutations identified in this study was predicted with the software PolyPhen-2 at http://genetics.bwh.harvard.edu/pph2/, and a mutation is classified as “probably damaging” if its probabilistic score is above 0.85 while as “possibly damaging” with the score above 0.15 [46].

The frequency of 1 novel splice-site and 4 missense mutations in at least 50 control individuals was investigated by newly-developed PCR-RFLP approaches. The PCR primer pairs were Ex-4F: 5′-TGGACAGACCACAATTCATCAA-3′ and IVS5B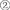:5′-GACGGAGTCTCGCTCTTTCA-3′, IVS5NF: 5′-TGAGGGCTTGTTAGATCAAGAT-3′ and IVS6NB:5′-TTACCCAGACAACAAATTAACCT-3′, IVS1NF:5′-TTTATGCACTGGGGCAACATG-3′ and Ex2Bm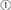:5′-TGCTCTCTTGGTTAAAGCCACT-3′, IVS13F:5′-GGATGTCACAGGCAGAGTTC-3′ and IVS14B:5′-CTCATCTGCCAGAATGAAGATT-3′, and IVS10F:5′-GGACTGATGCGGCTGTTAGA-3′ and SLCE12A:5′-TGGTGAGTTCCCCTGCTTTC-3′. The restriction endonucleases (REs) used for the RFLP analyses were Hin1 II (Fermentas), BseNI (Fermentas), PsiI (New England Biolabs), Taq I (Fermentas) and Hae III (Takara) for RFLP analysis, respectively. And, the frequency of the remaining missense mutations was explored by using direct DNA sequencing due to the lack of REs for their PCR-RFLP analysis. If parental DNA samples were available in some families, mutation co-segregation analyses were conducted by the PCR-RFLP or DNA sequencing approach described above. Moreover, by using a comparative alignment software of Genetyx® version 7.1 (Genetyx company, Tokyo, Japan), the amino acid sequences of human citrin and aralar were aligned with those in the homologous proteins from 9 different eukaryotic species, including chimpanzee, dog, mouse, rat, chicken, xenopus tropicalis, caenorhabditis elegans, opossum and cow. The amino acid sequences for the proteins were collected from the database ENSEMBL at http://www.ensembl.org/index.html.

### Total RNA Extraction and RT-nested PCR

In the patient C0054, only one paternally-inherited c.1399C>T mutation was revealed by routine screening and direct sequencing. The maternally-inherited mutation was further explored by mRNA analysis. Extraction of the total RNA and RT-nested PCR were performed as previously described [14], [33]. Briefly, PBLs were collected from heparinized venous blood of the patient, and the total RNA was extracted with RNAiso Plus (Takara). After that, 1 µg of RNA product was reverse-transcribed in the presence of 1 µg of oligo-(dT)18 and 200 U MMLV reverse transcriptase (Promega), and nested PCR was then performed with two pairs of primers, i.e. RAS2: 5′-AACGCACGCTGCCTGGCCGTATC-3′ and RACEA1: 5′-CCACCTTC ACAAATTCATGCGCC-3′ for the first PCR, while RAS3: 5′-GCCGCCGGGACTAGAAGTGAGC-3′ and Ex18R: 5′-TGCTTCATTCCCAGGAGGGA-3′ for the second. The amplicon contained the entire *SLC25A13* ORF, with a deduced size of 2191 bp.

### cDNA Cloning Analysis

After the mRNA extraction and RT-nested PCR, cDNA cloning was carried out in the patient C0054 to identify the aberrant mRNA molecule originated from the maternal allele that harbored an unknown mutation. In accord with the approach that was developed very recently [14], the nested PCR products were then separated by electrophoresis and the fragments of interest were excised and purified by gel extraction kit (Omega). Then the purified cDNA products were cloned into pSIMPLE-18 *Eco*R V/BAP Vector (Takara) and transformed into DH5α *Escherichia coli* competent cells by means of heat shock. The positive clones were screened by means of “white-blue spot selection” and tested by PCR with the primer pair RAS3 and Ex18R. The positive clones were then sequenced and comparatively aligned with the *SLC25A13* mRNA sequence. The alternative splicing variants (ASVs) identified by cDNA cloning analysis were described based on the nomenclature guidelines [47]. Chi square test was adopted to compare the proportion difference of the ASVs in the patient C0054 and 8 healthy volunteers, with *P*<0.05 as the significant criterion.

### SNP Analysis

The cDNA cloning analysis in the patient C0054 identified an aberrant transcript that was strongly suggestive of a large insertion/deletion mutation within the DNA fragment spanning from intron 4 to intron 5 in the *SLC25A13* gene. Subsequently, seven SNPs within this span, i.e. rs6465494(A/G), rs4727337(A/G) and rs67843496 (AA/−) in intron 4, and rs6943325(C/T), rs13307036(C/T), rs6465490 (C/A) and rs4273779(G/A) in intron 5, were analyzed by means of PCR amplification and direct sequencing. DNA fragments with heterozygous SNPs were not suspected to carry large insertion/deletion. On the contrary, if an amplicon was found with a homozygous SNP, a DNA fragment larger than the amplicon in size would be amplified by Long-range PCR (LA-PCR) using Takara LA Taq™ polymerase (Takara) according to the manufacturer’s instructions. The product of unexpected size was excised, purified and sequenced to explicit the nature of the likely large insertion/deletion in the patient C0054. The large transposal insertion identified in this patient was then deposited in GenBank (Accession number: KF425758).

### Update of the *SLC25A13* Mutations

It was mainly by using Pubmed, the well-recognized information retrieval system that comprises more than 22 million citations for biomedical literature from MEDLINE, life science journals, and online books (http://www.ncbi.nlm.nih.gov/pubmed/), that we collected all the references on CD since the year 1999 when the *SLC25A13* gene was cloned. The *SLC25A13* mutations identified in CTLN2 or NICCD patients, including their locations, types, and the DNA, cDNA and amino acid changes were updated and listed into a table.

### Mutation Spectrum in the Large Pediatric Cohort in China

The genotypic and phenotypic features of 60 new NICCD patients in China were described in this study. These new patients, along with the 56 subjects reported previously by our group [13], [14], [17], [33], [48], constituted a large Pediatric cohort of 116 CD patients in China. The *SLC25A13* mutation spectrum in this cohort was then summarized, and the proportion of every mutation in the whole spectrum was calculated and listed into a table. By using Chi square test and *P*<0.05 as the significant criterion, the distribution feature of *SLC25A13* mutations in South and North China were compared, with the latitude of 30°N as the dividing line, the most likely boundary between northern and southern Chinese who originated from distinct ancestors [49].

## Results

### Novel Point/Deletion Mutations

As shown in Table 1, 15 novel mutations, including 9 missense (c.443A>G, c.1498T>G, c.527G>T, c.1048G>A, c.1063C>G, c.415G>A, c.1364G>T, c.1215G>T and c.1775A>C), 4 nonsense (c.448G>T, c.1736G>A, c.1645C>T and c.72T>A), 1 splice-site (c.16-2A>T) and 1 deletion (c.265delG), were identified by direct DNA sequencing analysis of the 18 exons and their flanking sequences in *SLC25A13* gene of the 15 CD patients in East Asia, everyone of whom harbored a previously-reported mutation that had been uncovered by routine mutation screening. Figure S1 demonstrated the segmental sequencing results of the 15 mutations.

**Table 1:** Fifteen novel mutations in the *SLC25A13* gene of citrin-deficient patients from different countries.

Patient	Gender	Origin	Phenotype	SLC25A13 Genotypes			Effects	Frequency
				Patient	Father	Mother		
P111	Male	Japan	CTLN2	**c.448G>T**/IVS11+1G>A	–	–	p.E150X	–
P530	Male	Japan	NICCD	**c.1736G>A**/IVS16ins3kb	–	–	p.W579X	–
P645	Female	Japan	CTLN2	**c.443A>G**/c.851_854del	–	–	p.Y148C	<1%^△^
P874	Female	Japan	NICCD	**c.1498T>G**/IVS11+1G>A	IVS11+1G>A/N	c.1498T>G/N	p.Y500D	<1%^△^
P917	Female	Japan	NICCD	**c.16-2A>T**/IVS11+1G>A	IVS11+1G>A/N	c.16-2A>T/N	IVS1-2A>T	<1%^△^
P1075	Male	Japan	NICCD	**c.527G>T**/c.674C>A	–	–	p.G176V	<1%^△^
P1508	Male	Japan	CTLN2	**c.1645C>T**/c.674C>A	c.674C>A/N	c.1645C>T/N	p.Q549X	–
C0060	Male	China	NICCD	**c.72T>A**/c.775C>T	c.775C>T/N	c.72A>T/N	p.Y24X	–
C0079	Male	China	NICCD	**c.1048G>A**/IVS16ins3kb	IVS16ins3kb/N	c.1048G>A/N	p.D350N	<1%^▴^
C0098	Male	China	NICCD	**c.1063C>G**/c.1638_1660dup	c.1638_1660dup/N	c.1063C>G/N	p.R355G	<1%^▴^
C0106	Male	Malaysia	NICCD	**c.415G>A**/IVS16ins3kb	–	–	p.G139R	<1%^▴^
C0108	Female	China	NICCD	**c.1364G>T**/c.1638_1660dup	c.1364G>T/N	c.1638_1660dup/N	p.R455L	<1%^▴^
C0157	Male	China	NICCD	**c.265delG**/c.1638_1660dup	c.1638_1660dup/N	c.265delG/N	p.D89fs94X	–
C0168	Male	China	NICCD	**c.1215G>T/**c.851_854del	c.1215G>T/N	c.851_854del/N	p.K405N	<1%^△^
C0170	Female	China	NICCD	**c.1775A>C/**c.851_854del	c.851_854del/N	c.1775A>C/N	p.Q592P	<1%^▴^

The bold black letters denote the novel mutations, “N” indicates the normal SLC25A13 allele, while “–” means not analyzed. The effects and frequency refer to the corresponding novel mutations. The frequency was calculated based on the screening result by PCR-RFLP (**^△^**) or direct DNA sequencing approach (^▴^), respectively.

### Pathogenicity

**Figure 1:**
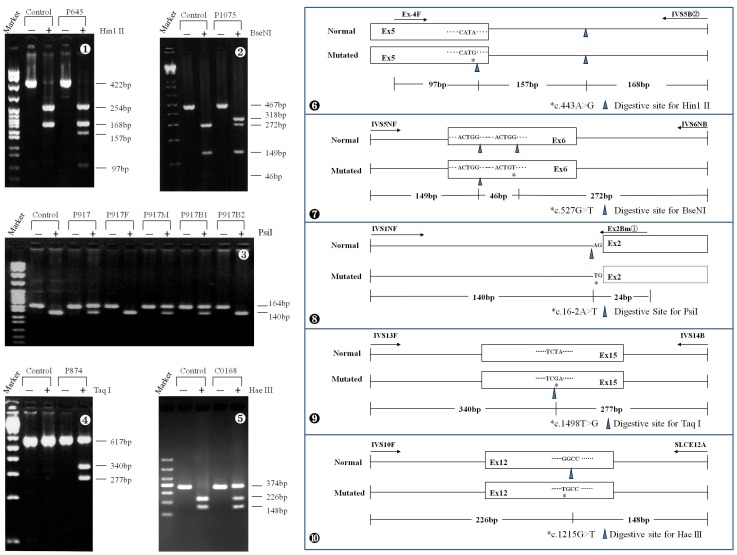
PCR-RFLP approaches for the carrier rate investigation of the 5 novel missense mutations. The figures 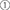 to 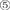 were representative gel electrophoresis of the RE-digested PCR products of the mutations c.443A>G, c.527G>T, c.16-2A>T, c.1498T>G and c.1215G>T, while the figures 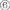 to 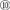 illustrated schematically the PCR-RFLP procedures for the 5 mutations, respectively. In 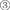, both the patient P917 and one of her brothers B1 harbored the maternally-inherited novel mutation c.16-2A>T.

**Figure 2:**
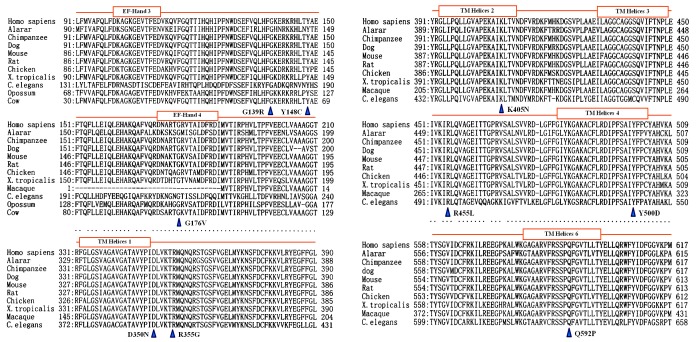
Comparative alignment of the homologous proteins. The homologous proteins include human citrin (Homo sapiens) and aralar, and the others from 9 different eukaryotic species, i.e. Chimpanzee, Dog, Mouse, Rat, Chicken, Xenopus Tropicalis (X.Tropicalis), Caenorhabditis elegans (C. elegans), Opossum and Cow, respectively. The closed brown boxes in this figure represented the EF-hand or TM helices in citrin protein, as clarified in the reference by Kobayashi et al, 1999.

The frequency of the missense and splice-site mutations were investigated in controls by the newly-developed PCR-RFLP, as illustrated in Figure 1, or sequencing approach, and all of them proved less than 1% (Table 1). Moreover, on family linkage analysis, the novel mutations demonstrated different parental sources with the previously-reported mutations in the same family (Table 1). In addition, comparative alignment of the homologous proteins in different eukaryotic species with human citrin and aralar further documented the conservative properties of the amino acids involved by the 9 missense mutations (Figure 2). And, on PolyPhen2 analysis of their function effect, Q592P was “possibly damaging” with a score 0.529 whilst the remaining 8 ones “probably damaging”, all with the score above 0.99. These evidences documented that the novel splice-site/missense mutations identified in this study were all disease-causing.

### Results of cDNA Cloning Analysis

The cDNA cloning analysis using PBLs in the patient C0054 uncovered a variety of ASVs from the maternally-inherited *SLC25A13* allele, and it was noteworthy that, as shown in Table 2, 95.8% (23/24) of the ASVs demonstrated exon 5 skipping (r.329_468del). However, *SLC25A13* cDNA cloning analysis in 8 healthy volunteers documented that the ASV harboring *r*.329_468del only made up a proportion of 0.9% (1/116, as shown in Table S1). The difference between the two proportions proved significant, with χ^2^ = 119.7 and *P*<0.01 (Table S2). The common feature of exon 5 skipping in the maternally-inherited ASVs in the patient C0054 indicated that the unknown mutation of maternal origin had affected the splicing mechanism around exon 5, strongly suggestive of a large insertion or deletion within the DNA segment spanning from intron 4 to intron 5 in the *SLC25A13* allele.

**Table 2:** The *SLC25A13* ASVs detected by cDNA analysis in the patient C0054.

Alleles	Name		ASVs		Remarks	Clones	%
Maternally-inherited	M-01		*r*.213_468del		Exon 4,5 skipping	8	33.3
	M-02		*r*.70_468del		Exon 3,4,5 skipping	4	16.7
	M-03		*r*.213_468del; *r*.1453_1591del		Exon 4,5,15 skipping	3	12.5
	M-04		*r*.329_468del		Exon 5 skipping	2	8.3
	M-05		*r*.16_69del; *r*.213_468del		Exon 2,4,5 skipping	1	4.2
	M-06		*r*.70_468del; *r*.69_469ins212+6499_212+6611;*r*.1018_1019ins1018+1_1018+469		Exon 3,4,5 skipping with intron3 and intron10fragment retention	1	4.2
	M-07		*r*.70_468del; *r*.1452_1453ins1452+12639_1452+12773		Exon 3,4,5 skipping with intron14 fragment retention	1	4.2
	M-08		*r*.70_468del; *r*.1750_1751ins1750+1_1750+93		Exon 3,4,5 skipping with intron16 retention	1	4.2
	M-09		*r*.213_468del; *r*.755_848del		Exon 4,5,8 skipping	1	4.2
	M-10		*r*.213_468del; *r*.69_70ins69+12147_69+12282		Exon 4,5 skipping with intron2 fragment retention	1	4.2
	M-11		*r*.213_328del		Exon 4 skipping	1	4.2
	In total					24	100
Paternally-inherited	P-01	*r*.213_328del; *r*.1399C>T		Exon 4 skipping		1	33.3
	P-02	*r*.213_468del; *r*.1399C>T		Exon 4,5 skipping		1	33.3
	P-03	*r*.213_328del;*r*.755_848del;*r*.1399C>T; *r*.1750_1751ins1750+1_1750+93		Exon 4,8 skipping with intron16 retention		1	33.3
	In total					3	100

In this table, all the maternally-inherited ASVs but M-11 harbored *r*.329_468del. The nucleotide numbering was based on *SLC25A13* cDNA sequence (GenBank: NM_014251), with +1 indicating the A of the ATG-translation initiation codon.

### Identification of a Large Transposal Insertion

**Figure 3:**
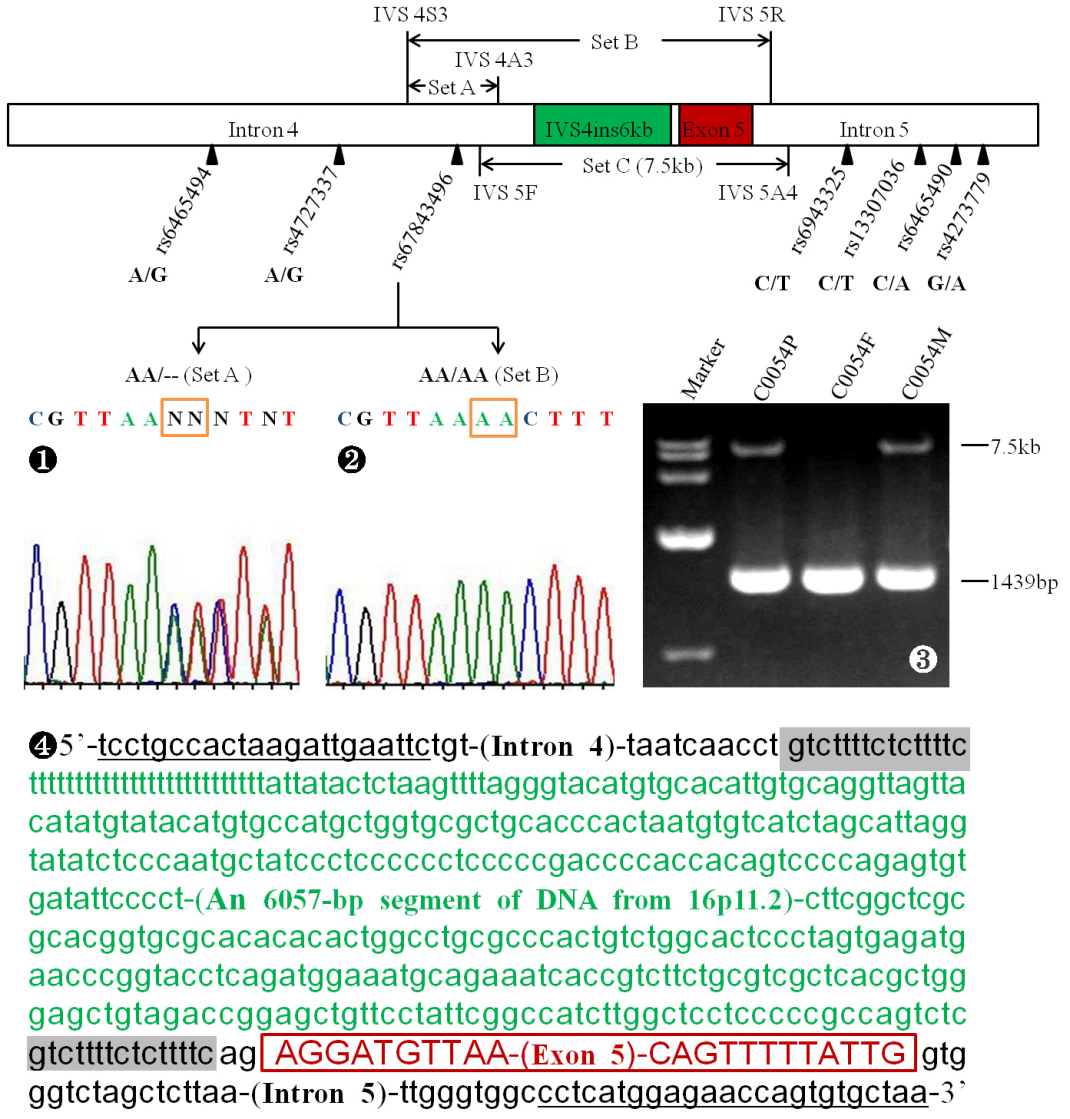
Identification of the large transposal insertion IVS4ins6kb (GenBank accession number: KF425758). Seven SNPs within the introns 4 and 5 were analyzed, and all of them were heterozygous but rs67843496 (The schematic overhead), an SNP detected heterozygously when amplified by the primers set A (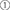) while homozygously with set B (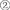). LA-PCR with the primers set C yielded an unexpected band of 7.5 kb inherited from the mother C0054M besides the expected 1439 bp product from the father C0054F (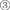). Segmental sequencing of the 7.5 bp product revealed a 6057 bp insertion from 16p11.2 (The sequence in green) along with two repetitive sequences of 15 bp at both sides (Shaded boxes), as illustrated in 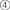. Underlined were the positions of the primers IVS5F and IVS5A4 (Set C) for LA-PCR.

Based on the cDNA cloning results, SNPs analysis was performed within the above DNA span of interest. The SNPs rs6465494, rs4727337, rs6943325, rs13307036, rs6465490 and rs4273779, as illustrated in Figure 3, all demonstrated heterozygous status. However, rs67843496 was heterozygous in the PCR product amplified with the primers set A, but homozygous with set B. This finding led to the design of the primers set C, and LA-PCR amplification with this primer set yielded an unexpected product of 7.5 kb of maternal origin, which was proved by segmental sequencing to be a transposal insertion of 6072 bp in length (IVS4ins6 kb, GenBank accession number: KF425758), with two repetitive sequences of 15 bp on the both sides of a 6057 bp large insertion translocated from chromosome 16p11.2. One of the two repetitive sequences is the fixed DNA sequence within intron 4.

### Update of *SLC25A13* Mutations

To date, a total of 81 *SLC25A13* sequence variations in CD patients have been reported worldwide (Table 3). The variations in the list demonstrated marked heterogeneity, comprising 32 missense (including 3 neutral), 18 nonsense, 14 splice-site (including c.1311C>T), 9 deletion, 3 insertion, 2 duplication, 1 indel (g./c.1610–1612delTAGinsAT), 1 aberrant transcript (*r*.16_212dup) with unknown mutation, and 1 pathogenic SNP (c.2T>C). Most (63%, 51/81) of the variations involved the C-terminal half of citrin protein that covers the 6 domains of transmembrane (TM) helices. In particular, 20 mutations were located within a DNA segment of 344 bp in size, spanning from exon 16 to exon 17 of the *SLC25A13* gene.

**Table 3:** Update of *SLC25A13* mutations in patients with citrin deficiency.

No.	Locations	Mutations	Types	DNA/cDNA changes	Amino acids	References
1	Ex1	c.2T>C	Pathogenic SNP	g./c.2T>C	p.Met1_Phe34del	14,34
2	Ex1	g.Ex1-1G>A	Splice-site	g./c.15G>A	Unclear	22
3	IVS1	IVS1-2A>T	Splice-site	g.16-2A>T	Unclear	This report
4	Ex2	E16X	Nonsense	g./c.46G>T	p.E16X	22
5	Ex3	Y24X	Nonsense	g./c.72T>A	p.Y24X	This report
6	Ex3	p.A25E	Missense	g./c.74C>A	p.A25E	21
7	Ex3	p.R43X	Nonsense	g./c.127C>T	p.R43X	21
8	Ex3	c.172_173delGT	Deletion	g./c.172_173delGT	p.V58Gfs81X	21
9	IVS2_IVS3	g./c.70-862_212+3527del4532	Deletion	c.70_212del	Unclear	20,35
10	Ex2_3	Unknown	Aberrant transcript	r.16_212dup (Ex2_3dup)	Unclear	14
11	Ex4	L85P	Missense	g./c.254T>C	p.L85P	23
12	Ex4	c.265delG	Deletion	g./c.265delG	p.D89fs94X	This report
13	IVS4	IVS4ins6kb	Insertion	r.329_468del(Ex5 del)	p.E110fs127X	This report
14	Ex5	G139R	Missense	g./c.415G>A	p.G139R	This report
15	Ex5	Y148C	Missense	g./c.443A>G	p.Y148C	This report
16	Ex5	E150X	Nonsense	g./c.448G>T	p.E150X	This report
17	Ex6	Q159X	Nonsense	g./c.475C>T	p.Q159X	31
18	Ex6	G176V	Missense	g./c.527G>T	p.G176V	This report
19	Ex6	R184X	Nonsense	g./(c.)550C>T	p.R184X	44
20	IVS6	g.IVS6+1G>C	Splice-site	c.IVS6(1789 bp)ins	p.A206fs212X	44
21	IVS6	IVS6+1G>A	Splice-site	g./c.615+1G>A	Unclear	23
22	IVS6	g.IVS6+5G>A	Splice-site	c. not detectable	Unclear	44
23	Ex7	S225X	Nonsense	g./c.674C>A	p.S225X	1
24	Ex7	Ex7-1G>A	Splice-site	g./c.754G>A/r.616_848del	p.A206fs213X	33
25	IVS7	IVS7-2A>G	Splice-site	g./c.755-2A>G	Unclear	28
26	Ex8	Q259X	Nonsense	g./c.775C>T	p.Q259X	32
27	Ex8	G283X	Nonsense	g./c.847G>T	p.G283X	13
28	IVS8	g.IVS8+3A>C	Splice-site	g./c.848+3A>C	Unclear	35
29	Ex9	851del4	Deletion	g./c.851_854delGTAT	p.R284fs286X	1
30	Ex9	Ex9-1G>A	Splice-site	c.933G>A	Unclear	16
31	Ex10	R319X	Nonsense	g./c.955C>T	p.R319X	28
32	Ex10	G333D	Missense	g./c.998G>A	p.G333D	17
33	Ex11	D350N	Missense	g./c.1048G>A	p.D350N	This report
34	Ex11	R355G	Missense	g./c.1063C>G	p.R355G	This report
35	Ex11	p.R355X	Nonsense	g./c.1063C>T	p.R355X	21
36	Ex11	R360X	Nonsense	g./c.1078C>T	p.R360X	22
37	Ex11	1092_1095delT	Deletion	g./c.1092_1095delT	p.F365fs407X	23
38	Ex11	1146delA	Deletion	g./c.1146delA	p.R383fs407X	22
39	Ex11	G386V	Missense	g./c.1157G>T	p.G386V	30
40	Ex11	G393S	Missense	g./(c.)1177G>A	p.G393S	27
41	IVS11	g.IVS11+1G>A	Splice-site	c.1019_1177del	p.340_392del	1
42	Ex12	Q397X	Nonsense	g./c.1189C>T	p.Q397X	26
43	Ex12	1192-1193delT	Deletion	g./c.1192_1193delT	p.L398fs407X	30
44	Ex12	c.1215G>T	Missense	g./c.1215G>T	p.K405N	This report
45	Ex13	c.1231G>A	Missense	g./c.1231G>A	p.V411M	13
46	Ex13	G436E	Missense	g./c.1307-1308delGCinsAA	p.G436E	38
47	Ex13	c.1311C>T	Splice-site?	g./c.1311C>T	p.C437C	32
48	IVS13	g.IVS13+1G>A	Splice-site	c.1231_1311del	p.411_437del	1
49	IVS13	g.IVS13+2T>G	Splice-site	c. not detectable	Unclear	22
50	Ex14	T446P	Missense	g./c.1336A>C	p.T446P	22
51	Ex14	K453R	Missense	g./c.1358A>G	p.K453R	30
52	Ex14	R455L	Missense	g./c.1364G>T	p.R455L	This report
53	Ex14	1374_1375delG	Deletion	g./c.1374 or 1375delG	p.A459fs507X	22
54	Ex14	R467X	Nonsense	g./c.1399C>T	p.R467X	30
55	IVS14_15	g.Ex15dup	Duplication	c.1453_1591dup	p.M532fs560X	19
56	Ex15	C489R	Missense	g./c.T1465C	p.C489R	37
57	Ex15	D493G	Missense	g./c.1478A>G	p.D493G	43
58	Ex15	Y500D	Missense	g./c.1498T>G	p.Y500D	This report
59	Ex15	P502L	Neutral	g./c.1505C>T	p.P502L	32,34
60	IVS15	g.IVS15+1G>T	Splice-site	c.1453_1591del	p.G485fs491X	22
61	Ex16	Q549X	Nonsense	g./c.1645C>T	p.Q549X	This report
62	Ex16	W579X	Nonsense	g./c.1736G>A	p.W579X	This report
63	Ex16	G531D	Missense	g.1592G>A	p.G531D	22
64	Ex16	c.1610_1612delTAGinsAT	Indel	g./c.1610_1612delTAGinsAT	p.L537fs538X	36
65	Ex16	A541D	Missense	g./c.1622C>A	p.A541D	28
66	Ex16	p.T546R	Missense	g./c.1637C>G	p.T546R	21
67	Ex16	T546M	Missense	g./c.1637C>T	p.T546M	22
68	Ex16	1638ins23	Duplication	g./c.1638_1660dup	p.A554fs570X	1
69	Ex16	R553Q	Missense	g./c.1658G>A	p.R553Q	31
70	Ex16_IVS17	g.Ex16+74_IVS17-32del516	Deletion	c. aberrant RNA	p.Q556fs565X	40
71	IVS16	g.IVS16ins3kb	Insertion	c. aberrant RNA	p.A584fs585X	22
72	Ex17	R588Q	Missense	g./c.1763G>A	p.R588Q	22
73	Ex17	L598R	Missense	g./c.1793T>G	p.L598R	25
74	Ex17	R585H	Missense	g./c.1754G>A	p.R585H	23
75	Ex17	Q592P	Missense	g./c.1775A>C	p.Q592P	This report
76	Ex17	1800ins1	Insertion	g./c.1799–1800insA	p.Y600X	39
77	Ex17	R605Q	Neutral	c.1814G>A	p.R605Q	34
78	Ex17	R605X	Nonsense	g./c.1813C>T	p.R605X	39
79	Ex17	E601X	Nonsense	g./(c.)1801G>T	p.E601X	8
80	Ex17	E601K	Missense	g./(c.)1801G>A	p.E601K	8
81	Ex18	P632L	Neutral	g./c.1895C>T	p.P632L	22,34

### New Chinese Patients with NICCD

We reported 60 new CD patients in this study (Table 4). All the 60 novel cases presented with clinical features of NICCD at the point of referral to our hospital. During their most recent follow-up, the NICCD manifestations had improved in 22 patients below 1 year of age. Among the 38 subjects beyond 1 year of age, 11 cases were healthy, without any laboratory or clinical abnormalities. However, hepatomegaly was found in 1 patient, and anemia in 2 cases. Dyslipidemia was revealed in 7 cases while failure to thrive (FTT) in 3, and in particular, 5 patients had FTT and dyslipidemia concurrently, constituting 5 additional patients with FTTDCD features. The remaining 9 patients were lost in contact.

**Table 4:** Molecular and clinical information of 60 new NICCD patients in China.

Case	Patient	Gender	Mutations	Major presentations	Clinical outcomes
57	C0049	Male	851del4/851del4	NICCD	Dyslipidemia
58	C0050	Female	851del4/851del4	NICCD	FTTDCD
59	C0054	Male	R467X/IVS4ins6kb	NICCD	Dyslipidemia
60	C0056	Male	851del4/851del4	NICCD, ASD+VSD	α-Thalassemia
61	C0057	Male	851del4/1638-1660dup	NICCD	Normal
62	C0060	Male	Q259X/c.72A>T	NICCD	Dyslipidemia
63	C0063	Male	851del4/851del4	NICCD	FTTDCD
64	C0068	Male	851del4/851del4	NICCD	Dyslipidemia
65	C0069	Male	851del4/IVS6+5G>A	NICCD	Lost contact
66	C0070	Female	851del4/851del4	NICCD	Lost contact
67	C0071	Female	851del4/851del4	NICCD	Dyslipidemia
68	C0073	Female	851del4/851del4	NICCD	Lost contact
69	C0075	Female	851del4/851del4	NICCD	Lost contact
70	C0076	Female	851del4/1638-1660dup	NICCD	FTTDCD
71	C0078	Male	851del4/851del4	NICCD	Normal
72	C0079	Male	IVS16ins3kb/c.1048G>A	NICCD	Lost contact
73	C0082	Male	1638-1660dup/1638-1660dup	NICCD	Lost contact
74	C0086	Male	851del4/851del4	NICCD	Lost contact
75	C0087	Male	851del4/1638-1660dup	NICCD	Dyslipidemia
76	C0095	Male	851del4/IVS6+5G>A	NICCD	Normal
77	C0097	Male	851del4/851del4	NICCD	Hepatomegaly
78	C0098	Male	1638-1660dup/c.1063C>G	NICCD	Lost contact
79	C0099	Memale	851del4/IVS6+5G>A	NICCD	Normal
80	C0108	Female	1638-1660dup/c.1364G>T	NICCD	Normal
81	C0109	Male	851del4/851del4	NICCD	Normal
82	C0110	Female	IVS6+5G>A/R319X	NICCD	Normal
83	C0116	Male	IVS6+5G>A/IVS16ins3kb	NICCD	Normal
84	C0123	Female	851del4/IVS16ins3kb	NICCD, liver cirrhosis	FTTDCD, ascites, umbilical hernia, Hematochezia
85	C0124	Male	IVS6+5G>A/?	NICCD, eczema	Normal
86	C0125	Male	Q159X/R467X	NICCD	Dyslipidemia
87	C0129	Male	851del4/IVS6+5G>A	NICCD, liver cirrhosis	FTTDCD, Died of liver cirrhosis at his age of 2 years and 5 months
88	C0130	Male	851del4/851del4	NICCD, Iron-deficient anemia	Iron-deficient anemia
89	C0134	Male	851del4/IVS6+5G>A	NICCD, cerebral palsy	FTT, improved cerebral palsy
90	C0136	Female	851del4/R467X	NICCD	Normal
91	C0139	Female	1092-1095delT/c.754G>A	NICCD	FTT
92	C0139B	Male	1092-1095delT/c.754G>A	NICCD	Normal
93	C0140	Female	IVS11+1G>A/IVS16ins3kb	NICCD	Improved cholestasis
94	C0142	Female	851del4/851del4	NICCD	Improved cholestasis
95	C0143	Male	851del4/851del4	NICCD	Improved cholestasis
96	C0144	Female	851del4/851del4	NICCD	Normal
97	C0146	Male	IVS16ins3kb/IVS16ins3kb	NICCD	FTT
98	C0147	Male	1638ins23/IVS6+5G>A	NICCD	Improved cholestasis
99	C0068S	Female	851del4/851del4	NICCD	Lost contact
100	C0148	Female	851del4/851del4	NICCD	Improved cholestasis
101	C0149	Female	851del4/851del4	NICCD	Improved cholestasis
102	C0150	Male	IVS6+5G>A/R319X	NICCD	Improved cholestasis
103	C0151	Female	851del4/?	NICCD	Improved cholestasis
104	C0154	Female	851del4/851del4	NICCD	Improved cholestasis
105	C0156	Female	851del4/IVS16ins3kb	NICCD	Improved cholestasis
106	C0157	Male	1638ins23/c.265delG	NICCD	Improved cholestasis, hydrocele
107	C0158	Female	851del4/IVS16ins3kb	NICCD	Improved cholestasis, congenital muscular torticollis, inguinal hernia
108	C0159	Male	851del4/851del4	NICCD	Improved cholestasis
109	C0160	Female	851del4/IVS16ins3kb	NICCD	Improved cholestasis
110	C0161	Female	851del4/1092-1095delT	NICCD	Improved cholestasis
111	C0162	Female	851del4/851del4	NICCD	Improved cholestasis
112	C0163	Female	851del4/851del4	NICCD	Improved cholestasis
113	C0166	Male	1638ins23/1638ins23	NICCD	Improved cholestasis
114	C0168	Male	851del4/c.1215G>T	NICCD	Improved cholestasis
115	C0169	Female	851del4/?	NICCD	Improved cholestasis
116	C0170	Female	851del4/c.1775A>C	NICCD	Improved cholestasis

### Demographic Features of the Chinese Pediatric Cohort

**Figure 4:**
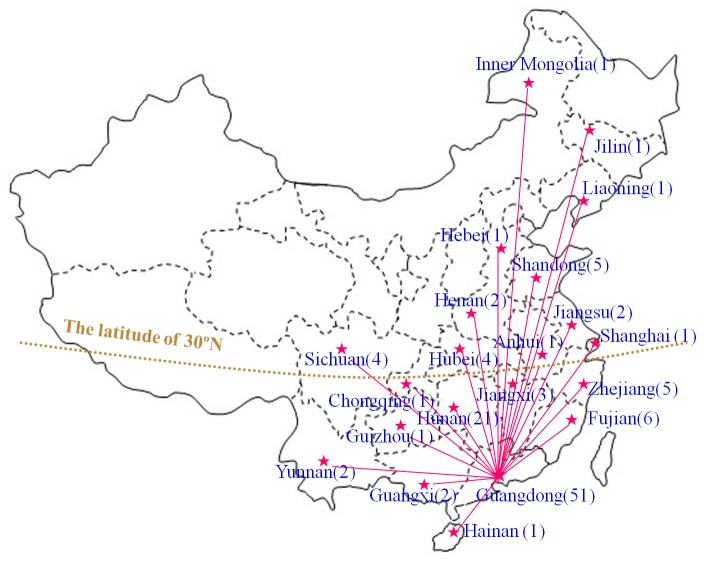
Native places of the 116 Chinese patients with citrin deficiency. By the end of February in 2013, 116 Chinese patients from 21 provinces, municipalities and autonomous regions in China were diagnosed by *SLC25A13* gene analysis by our group. This figure indicated their native places, with the patient numbers in the parentheses behind.

So far, 116 Chinese CD patients have been diagnosed by our group. This pediatric cohort consisted of 48 females and 68 males, and involved 113 families from 21 provinces, municipalities and autonomous regions in China, including Guangdong, Guangxi, Hainan, Hunan, Hubei, Yunnan, Guizhou, Sichuan, Chongqing, Jiangxi, Fujian, Zhejiang, Jiangsu, Anhui, Shanghai, Henan, Hebei, Shandong, Liaoning, Jilin and Inner Mongolia. As shown in Figure 4, 93 out of the 116 patients were from South China, especially from Guangdong province (51 patients).

### The Distribution of *SLC25A13* Mutations in the Chinese Pediatric Cohort

As shown in Table 5, the 4 mutations 851_854del4, 1638_1660dup, IVS6+5G>A and IVS16ins3kb were on the top of the list, and accounted for 83.19% of all mutated *SLC25A13* alleles, constituting the high-frequency mutations in this Chinese CD cohort. The remaining 22 mutations contributed 14.11%, whilst the unknown ones, just 2.7%. In South China, the 4 high-frequency mutations contributed 87.9% of the total mutated alleles, while this proportion in the North was just 63.6%, and this difference in the mutation distribution between the two areas in China was proved to be statistically significant, with χ^2^ = 14.93 and *P*<0.01, as shown in Table S3.

**Table 5:** Frequency and proportion of the mutated *SLC25A13* alleles in the large Chinese Pediatric cohort.

No.	Mutations	Frequency	Percentage
01	851_854del4	132	58.41
02	1638-1660dup	20	8.85
03	IVS6+5G>A	19	8.41
04	IVS16ins3kb	17	7.52
05	c.1399C>T(R467X)	5	2.21
06	c.955C>T(R319X)	3	1.33
07	IVS11+1G>A	3	1.33
08	c.754G>A	2	0.88
09	c.1092_1095delT	2	0.88
10	c.1078C>T(R360X)	1	0.44
11	c.1622C>A(A541D)	1	0.44
12	c.1231G>A(V411M)	1	0.44
13	c.847G>T(G283X)	1	0.44
14	c.998G>A(G333D)	1	0.44
15	c.475C>T(Q159X)	1	0.44
16	c.775C>T(Q259X)	1	0.44
17	g.2T>C	1	0.44
18	r.16-212dup	1	0.44
19	c.72T>A(Y24X)	1	0.44
20	c.1048G>A(D350N)	1	0.44
21	c.1063C>G(R355G)	1	0.44
22	c.1364G>T(R455L)	1	0.44
23	IVS4ins6kb	1	0.44
24	c.1215G>T(K405N)	1	0.44
25	c.265delG	1	0.44
26	c.1775A>C(Q592P)	1	0.44
27	Unknown	6	2.7
In total		226*	100

* Among the 116 patients, there were 3 siblings from 3 families. Therefore, the cohort comprised 226 mutated *SLC25A13* alleles in total, including 6 unknown ones.

## Discussion

Since *SLC25A13* was cloned as the causative gene for CD [1], genetic analysis of this gene had been well-recognized as a reliable tool for the definitive diagnosis of CD patients. By direct DNA sequencing, 15 novel *SLC25A13* variations were identified in this paper (Table 1 and Figure S1), and all of them proved to be CD-associated pathogenic mutations by laboratory and bioinformatic evidences (Table 1 and Figures 1 and 2). These novel mutations expanded the *SLC25A13* mutation spectrum, and provided conclusive genetic evidences for the definitive diagnosis of the East Asian patients, along with the mutations revealed at screening analysis. However, routine DNA analytic approaches such as PCR-RFLP and sequencing could not identify all *SLC25A13* mutations [13], [24], [44], and the patient C0054 in this paper was such a case, in whom a paternally-inherited c.1399C>T mutation was revealed by screening and direct sequencing, while the mutation of maternal origin once remained obscure on such routine DNA analytic tools.

Our recent cDNA cloning analysis of *SLC25A13* ASVs in human PBLs uncovered the marked transcript diversity along with the abundant existence of the transcript *r*.213_328del that predicted the existence of a constructively novel isoform for citrin protein. Similar ASVs could be detected for mutated *SLC25A13* alleles, but all of them carried the information of/from their corresponsive mutations [14]. *SLC25A13* cDNA analysis in this study (Table 2 and Table S1) confirmed our previous findings, and particularly, the unique feature of exon 5 skipping in the ASVs of maternal origin in the patient C0054 (Table S2) provided a reliable evidence for the positioning of a large insertion, or deletion, within the *SLC25A13* gene segment spanning from intron 4 to intron 5, and directly led to the final identification of the novel transposal insertion of IVS4ins6kb. These findings once again supported the concept that cDNA cloning analysis of *SLC25A13* gene using human PBLs could be taken as a feasible tool for the molecular diagnosis of citrin deficiency, overcoming the technical limitation of the conventional DNA analysis.

The large IVS4ins6kb mutation in this study is the second transposal insertion besides the one reported in 2008 [22]. This large insertion was identical in sequence to a DNA segment of 6057bp at chromosome 16p11.2, with two 15bp repetitive sequences on the both sides as the target-site duplication for this DNA transposon (Figure 3). We have no direct evidences yet to clarify the pathogenic mechanism underlying this transposal insertion. However, since IVS4ins6kb occurred very closely to the branch point site within intron 4, a reasonable explanation might be that the large insertion interrupted the formation of the lariat structure and thus disrupted the excision of intron 4 during the splicing reaction. However, this transposal insertion did not affect the branch point site within intron 5, and the freed 5′ end of intron 4 thus had to joined to this site alternatively, forming a lariat and giving rise to exon 5 skipping in the *SLC25A13* transcripts, as revealed by cDNA cloning analysis using PBLs in the patient C0054 (Table 2). The exon 5 skipping predictively resulted in a frameshift at codon 110, added 17 amino acids, and then introduced a stop codon at position 127, thus yielding a truncated citrin molecule p.E110fs127X.

To the best of our knowledge, Table 3 was the most comprehensive update of the reported *SLC25A13* variations up to now, and the heterogenetic variations in this table constituted reliable molecular evidences for the definitive diagnosis of CD patients. However, it should be recognized that the list itself remained far from being perfect currently. One issue was the unclear mechanisms underlining specific mutations. For example, c.1311C>T had been reported as a synonymous mutation p.C437C [32], but this C>T substitution might cause abnormal splicing of pre-mRNA since it involved the last base of the exon 13 in *SLC25A13* gene. Another issue lied in the limited experience on citrin protein analysis. Although the aberrant transcript *r*.16_212dup had been identified in a NICCD patient [14], its biochemical and structural effects on citrin protein remained obscure due to the technical limitation. The pathogenicity of some missense mutations was another issue. Actually, AGC2 function analysis had documented that c.1505C>T, c.1814G>A and c.1895C>T were all neutral, but not missense mutations [34]. The above issues once again necessitated the in-depth molecular analysis of CD by means of not only genetic, but also transcriptional, translational, and even functional tools.

Although the number of reported Chinese CD patients was relatively small, as a country with a population over 1.3 billion, China might be the largest victim of CD, currently having about 85 700 of such patients theoretically. We reported 60 new NICCD patients in this study (Table 4), and thus established a Pediatric cohort of 116 CD cases, providing a foundation for the in-depth investigation of this disease entity in China. In the *SLC25A13* mutation spectrum, as shown in Table 5, 851_854del4, 1638_1660dup, IVS6+5G>A and IVS16ins3kb could be considered as high-frequency mutations in China while the remaining 22 mutations are mostly sporadic. This finding provided an important evidence for the screening of targeted *SLC25A13* mutations in Chinese population. The native place analysis of the CD patients in Figure 4 uncovered that most patients are originated from south China, which could be explained partially by the higher carrier rate of *SLC25A13* mutations in this area than in the north [44]. The geographic location of our hospital might be another likely reason. As the capital city of Guangdong province, Guangzhou was the largest city in South China.

The *SLC25A13* mutations were distributed differently in the patients from different areas in China, and as shown in Table S3, the 4 high-frequency mutations were more common in southern Chinese than in northern. This distribution difference might be attributed to the founder effect and genetic drift. The 4 high-frequency mutations had occurred earlier in southern Chinese during the long history of human evolution, and then dispersed into northern Chinese along with the population migration. Actually, haplotype analysis has proved that the mutation 851_854 del arose in the southern mongoloid population [44], who originated in the Guangxi and Yunnan areas in the southwest China [50]. On the other hand, it has been proposed that the contemporary northern and southern Chinese have distinct origin site in the Yellow River valley and the Yangtze River valley, respectively, with the most likely boundary drawn at the latitude of 30°N [51], and anthropologic evidences have suggested that, in the early Middle Pleistocene, the early man in south of the Yangtze River dispersed to the Qinling Mountain and south of the Yellow River [49].

In conclusion, the 16 novel pathogenic mutations identified in this study enriched the variation spectrum of *SLC25A13* gene, and the comprehensive update of *SLC25A13* mutations provided a reliable molecular evidence for the definitive diagnosis of CD patients. And, the establishment of the large Chinese Pediatric cohort, the *SLC25A13* mutations in this cohort, along with their distribution difference in the patients from different areas in China, formed a substantial contribution to the in-depth understanding of the genotypic features of Chinese CD patients.

## Supporting Information

Figure S1 **Direct DNA sequencing results of the point and deletion mutations in** ***SLC25A13*** **gene.** In this study, 1 deletion and 14 point mutations of *SLC25A13* gene were identified in 15 patients with citrin deficiency. Arrows were used in this figure to indicate the mutated bases with description overhead, respectively.
(TIF)

Table S1 **The** ***SLC25A13*** **ASVs detected by cDNA analysis in eight healthy volunteers.**
(DOC)

Table S2 **Comparison of the** ***SLC25A13*** **ASVs harboring** ***r*** **.329_468del in the patient C0054 and the 8 healthy volunteers.**
(DOC)

Table S3 **Distribution of the mutated** ***SLC25A13*** **alleles in the CD patients from South and North China.**
(DOC)
