# Supplementary figures and images for: SLC25A13 Gene Analysis in Citrin Deficiency: Sixteen Novel Mutations in East Asian Patients, and the Mutation Distribution in a Large Pediatric Cohort in China
Source: PLoS One. 2013 Sep 19;8(9):e74544. doi: 10.1371/journal.pone.0074544 (PMC3777997; doi:10.1371/journal.pone.0074544)

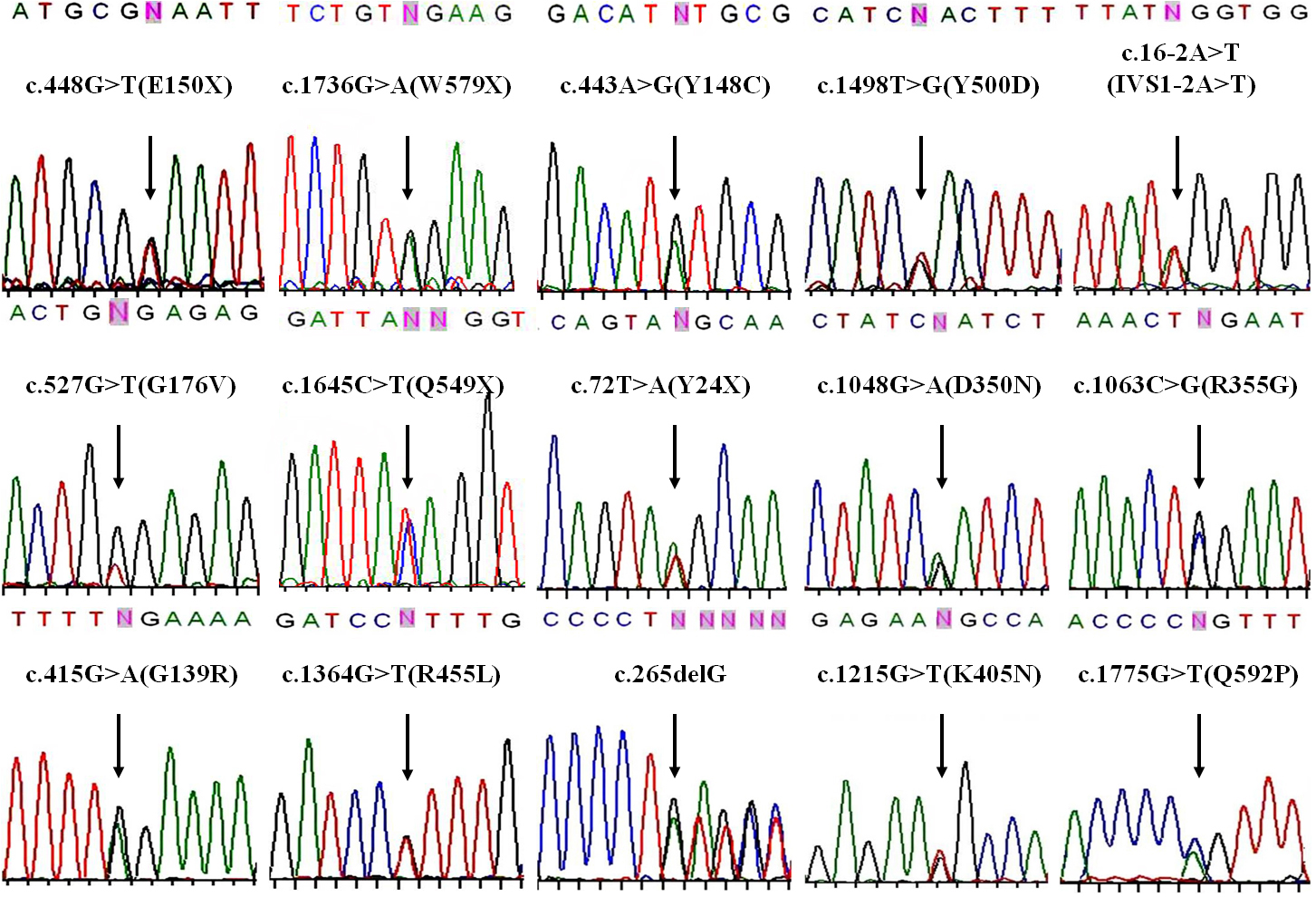

Supplement: Figure S1 — Direct DNA sequencing results of the point and deletion mutations in SLC25A13 gene. In this study, 1 deletion and 14 point mutations of SLC25A13 gene were identified in 15 patients with citrin deficiency. Arrows were used in this figure to indicate the mutated bases with description overhead, respectively. (TIF) [file pone.0074544.s001.tif]
