# Supplementary material for: SLC25A13 Gene Analysis in Citrin Deficiency: Sixteen Novel Mutations in East Asian Patients, and the Mutation Distribution in a Large Pediatric Cohort in China
Source: PLoS One. 2013 Sep 19;8(9):e74544. doi: 10.1371/journal.pone.0074544 (PMC3777997; doi:10.1371/journal.pone.0074544)
Supplement: Table S1 — The SLC25A13 ASVs detected by cDNA analysis in eight healthy volunteers. (DOC) [file pone.0074544.s002.doc]

**Table S1.** The *SLC25A13* ASVs detected by cDNA analysis in eight healthy volunteers

| **Name** | **ASVs** | **Remarks** | **Clones** | **%** |
| --- | --- | --- | --- | --- |
| C-01 | *r*.= | Normal | 31 | 26.7 |
| C-02 | *r*.213_328del | Exon 4 skipping | 44 | 37.9 |
| C-03 | *r*.755_848del | Exon 8 skipping | 1 | 0.9 |
| C-04 | *r*.70_328del | Exon 3, 4 skipping | 2 | 1.7 |
| C-05 | *r*.213_328del; *r*.755_848del | Exon 4, 8 skipping | 9 | 7.8 |
| C-06 | *r*.213_468del; *r*.755_848del | Exon 4, 5, 8 skipping | 1 | 0.9 |
| C-07 | *r*.1750_1751ins1750+1_1750+93 | Intron16 retention | 1 | 0.9 |
| C-08 | *r*.213_328del; *r*.1750_1751ins1750+1_1750+93 | Exon 4 skipping with intron16 retention | 5 | 4.3 |
| C-09 | *r*.755_848del; *r*.1750_1751ins1750+1_1750+93 | Exon 8 skipping with intron16 retention | 1 | 0.9 |
| C-10 | *r*.213_328del; *r*.755_933del; *r*.1018_1019ins1018+1_1018+491 | Exon 4, 8, 9 skipping with intron10 fragment retention | 3 | 2.6 |
| C-11 | *r*.1311_1312ins1311+102_1311+176 | Intron13 fragment retention | 3 | 2.6 |
| C-12 | *r*.213_328del; *r*.1452_1453ins1452+12639_1452+12758 | Exon 4 skipping with intron14 fragment retention | 1 | 0.9 |
| C-13 | *r*.69_70ins69+12147_69+12282; *r*.213_328del | Exon 4 skipping with intron2 fragment retention | 1 | 0.9 |
| C-14 | *r*.213_328del; *r*.1311_1312ins 1311+102_1311+176 | Exon 4 skipping with intron13 fragment retention | 3 | 2.6 |
| C-15 | *r*.755_848del; *r*.1311_1312ins 1311+102_1311+176 | Exon 8 skipping with intron13 fragment retention | 1 | 0.9 |
| C-16 | *r*.70_328del; *r*.1452_1453ins1452+12639_1452+12758 | Exon 3, 4 skipping with intron14 fragment retention | 4 | 3.4 |
| C-17 | *r*.212_213ins212+6499_212+6611; *r*.213_328del; r.468_469ins468+12874_468+12970; *r*.755_848del; *r*.993_1018del | Exon 4, 8 skipping with intron3 fragment retention, intron5 fragment retention and exon10 fragment deletion | 2 | 1.7 |
| C-18 | *r*.70_328del; *r*.212_213ins212+6499_212+6611; *r*.755_848del; *r*.1453_1591del | Exon 3, 4, 8, 15 skipping with intron3 fragment retention | 2 | 1.7 |
| C-19 | *r*.213_328del; *r*.755_933del | Exon 4, 8, 9 skipping | 1 | 0.9 |
| In Total | | | 116 | 100 |

In This table, C-06 was the only ASV harboring *r*.329_468del. The nucleotide numbering was based on *SLC25A13* cDNA sequence (GenBank: NM_014251), with +1 indicating the A of the ATG-translation initiation codon.
