# Supplementary material for: SLC25A13 Gene Analysis in Citrin Deficiency: Sixteen Novel Mutations in East Asian Patients, and the Mutation Distribution in a Large Pediatric Cohort in China
Source: PLoS One. 2013 Sep 19;8(9):e74544. doi: 10.1371/journal.pone.0074544 (PMC3777997; doi:10.1371/journal.pone.0074544)
Supplement: Table S2 — Comparison of the SLC25A13 ASVs harboring r .329_468del in the patient C0054 and the 8 healthy volunteers. (DOC) [file pone.0074544.s003.doc]

**Table S2.** Comparison of the *SLC25A13* ASVs harboring *r*.329_468del in the patient C0054 and the 8 healthy volunteers

| Subjects | *SLC25A13* ASVs | | χ^2^ | *P* |
| --- | --- | --- | --- | --- |
|  | with *r*.329_468del | without *r*.329_468del |  |  |
| Patient C0054 | 23 (95.8%) | 1 (4.2%) | 119.7 | <0.01 |
| Healthy volunteers | 1 (0.9%) | 115 (99.1%) |  |  |

Correction for Continuity was performed for χ^2^ calculation in this table.
