# Supplementary material for: SLC25A13 Gene Analysis in Citrin Deficiency: Sixteen Novel Mutations in East Asian Patients, and the Mutation Distribution in a Large Pediatric Cohort in China
Source: PLoS One. 2013 Sep 19;8(9):e74544. doi: 10.1371/journal.pone.0074544 (PMC3777997; doi:10.1371/journal.pone.0074544)
Supplement: Table S3 — Distribution of the mutated SLC25A13 alleles in the CD patients from South and North China. (DOC) [file pone.0074544.s004.doc]

**Table S3.** Distribution of the mutated *SLC25A13* alleles in the CD patients from South and North China

| Areas in China | Mutated *SLC25A13* alleles | | χ^2^ | *P* |
| --- | --- | --- | --- | --- |
|  | High-frequency | Non-high-frequency |  |  |
| North | 28 (63.6%) | 16 (36.4%) | 14.93 | <0.01 |
| South | 160 (87.9%) | 22 (12.1%) |  |  |

The numbers and proportions in this table referred to all the mutated *SLC25A13* alleles in Table 5.
